# The Induced Pluripotent Stem Cells in Articular Cartilage Regeneration and Disease Modelling: Are We Ready for Their Clinical Use?

**DOI:** 10.3390/cells11030529

**Published:** 2022-02-03

**Authors:** Michał S. Lach, Monika A. Rosochowicz, Magdalena Richter, Inga Jagiełło, Wiktoria M. Suchorska, Tomasz Trzeciak

**Affiliations:** 1 Radiobiology Laboratory, Greater Poland Cancer Centre, 61-866 Poznan, Poland; monika.rosochowicz@interia.eu; 2 Department of Orthopedics and Traumatology, Poznan University of Medical Sciences, 61-545 Poznan, Poland; mrichter@ump.edu.pl (M.R.); tomasz.trzeciak@ump.edu.pl (T.T.); 3 Department of Electroradiology, Poznan University of Medical Sciences, 61-866 Poznan, Poland; wiktoria.suchorska@wco.pl; 4 Greater Poland Cancer Centre, Department of Tumour Pathology, 61-866 Poznan, Poland; inga.jagiello@wco.pl

**Keywords:** iPSC, osteoarthritis, stem cells, disease modelling, chondrodysplasias, regenerative medicine

## Abstract

The development of induced pluripotent stem cells has brought unlimited possibilities to the field of regenerative medicine. This could be ideal for treating osteoarthritis and other skeletal diseases, because the current procedures tend to be short-term solutions. The usage of induced pluripotent stem cells in the cell-based regeneration of cartilage damages could replace or improve on the current techniques. The patient’s specific non-invasive collection of tissue for reprogramming purposes could also create a platform for drug screening and disease modelling for an overview of distinct skeletal abnormalities. In this review, we seek to summarise the latest achievements in the chondrogenic differentiation of pluripotent stem cells for regenerative purposes and disease modelling.

## 1. Introduction

Over a decade has passed since the Yamanaka group developed the reprogramming process, which revolutionised the usage of pluripotent stem cells in the field of regenerative medicine and created hitherto unimaginable possibilities. However, modifying the cell culture conditions to manipulate these cells toward the desired one remains challenging. Bioengineers and clinicians are looking for efficient cartilage regeneration methods to treat injuries and reduce the deterioration in the quality of life caused by osteoarthritis (OA) [1,2,3,4,5,6]. Soon, the distinct disabilities regarding the development of OA will gain the status of a civilisation disease due to their increasing percentage of the elderly population, especially in well-developed countries [5,7,8]. As osteoarthritis progresses, the biomechanical properties of the joint are altered, causing pain and stiffness and limiting the range of motion [8,9]. The lack of symptoms at the early stages of OA rules out non-invasive treatment to prevent further progress of the disease [5,10,11]. The development of OA is related to the remodelling of the articular cartilage extracellular matrix (ECM) caused by the matrix metalloproteinases (MMP) and disintegrins and metalloproteinases with thrombospondin motif (ADAMTS) [12].

The articular cartilage matrix is enriched with collagens (type IX, II, and XI); laminin; and elastin. Additionally incumbent are proteoglycans containing hyaluronic acid, chondroitin sulphate, and keratan sulphate [13,14,15]. The proportion of water in cartilage tissue (70–80%) also increases the resistance of cartilage to mechanical stress, forces ranging from 1 to 4 MPa, and facilitates joint movement [16,17]. The articular cartilage may distinguish three functional regions: the superficial, middle, and deep zones. The superficial zone is enriched with lubricin, hyaluronic acid, and flattened chondrocytes. These are responsible for sustaining homogenous joint surfaces and reducing the friction forces. The collagen fibres are also parallel to each other. The middle zone is composed of dense ECM with increased amounts of proteoglycans and is responsible for high osmotic pressure, the distribution of mechanical forces and resilience. There are randomly located spherical chondrocytes, and chaotically distributed fibres may be found. The deep zone is characterised by enlarged, mature chondrocytes, clearly expressing collagen type X, in which the fibres are oriented perpendicularly [18,19]. Due to the avascular nature of cartilage, a dense ECM enables the exchange of nutrition and waste products by diffusion from the synovial fluid and subchondral bone [14,20,21,22].

Therefore, new approaches are required to eliminate or prolong the useful life of the most invasive procedures, extending the patients’ quality of life and activity. This is the socioeconomic burden of ageing societies. The new technologies and discoveries in the field of regenerative medicine, especially regarding the usage of induced pluripotent stem cells, could yield a solution to the possible future increase in cases of disabilities caused by OA. These alternatives could improve or replace the existing surgical procedures, which have drawbacks (detailed in the next section).

In this review, we focus on the recent discoveries regarding the development of suitable differentiation protocols of human-induced pluripotent stem cells into chondrogenic populations and their possible role in clinic and disease modelling.

## 2. The Surgical Methods in OA Treatment

The standard therapeutic procedures of articular cartilage repair involve the regeneration of the cartilage with autologous chondrocyte implantation (ACI), matrix-induced autologous chondrocyte implantation (MACI), microfracture, mosaicplasty, and the most extensive option of total joint arthroplasty. These procedures have both advantages and disadvantages, summarised in Table 1.

Briefly, in the ACI, chondrocytes removed from the patient are propagated in vitro. They undergo dedifferentiation, reducing the expression of type II collagen and proteoglycans. The number of cells required for the repair procedure is obtained but represents a diminished functionality. There is a problem concerning the dedifferentiation of chondrocytes after implantation; hence, fibrocartilage formation is observed more than the expected hyaline cartilage [39,40,41,42]. Another limitation of this technique is the donor’s age, since the number of chondrocytes capable of proliferating and producing functional cartilage decreases with age [1].

MACI is a modified ACI procedure. The main difference between the techniques is the application of collagen matrices seeded with autologous chondrocytes. This resolves the problem of periosteal patch leakage, one of the side effects of ACI [13]. ACI and MACI result in patients’ relatively quick recovery after surgery [43]. Still, these current techniques have some severe disadvantages. The main problem is the scarcity of cells post-implantation. Chondrocytes constitute only 2% of the whole cartilage, which complicates the autotransplantation of cells and requires the damage of healthy tissue to obtain sufficient cells. These procedures are also performed on active young patients. Ultimately, it is not a long-term solution due to the formation of fibrocartilage in place of hyaline cartilage, which leads to further damage [26,44,45]. On the other hand, microfractures use the potential of bone marrow mesenchymal stem cells (MSCs) to separate into chondrocyte-like cells. However, the regenerated tissue shows fibrocartilage properties, which changes the biomechanical functions of the joint and yields unsatisfactory results in the long term [42,46]. The benefit of microfractures comes from their simplicity, low costs, and requiring a single-step surgical procedure [47]. 

These procedures may also treat substantial full-thickness defects using mosaicplasty or osteochondral autograft transplant (OAT) [48]. This procedure is quite simple and could be performed arthroscopically but is longer and more complex than a microfracture [49]. The osteochondral plug is collected from the low-bearing site of the joint and then placed in the damaged area. The graft is generally well-built-up in the surrounding bone but less efficient in the site of hyaline cartilage [50]. The cyclic pressure on the graft may mean that some loosening may occur in the long term, and more significant defects may develop, as with other techniques [23,48]. This procedure may have better outcomes than microfractures but fewer than ACI [47].

Even these techniques fail to rule out patients finally undergoing the most invasive procedure, i.e., joint arthroplasty [4,35]. This procedure reduces the pain and stiffness but also limits activity post-surgery. The costs of materials used for manufacturing implants are also high. After a couple of years, revision surgery is necessary to replace the worn implants, and this causes a higher risk of the development of septic or aseptic loosening of part of the implants [51].

## 3. iPS Cells Definition

Induced pluripotent stem cells (iPSC) are a specific type of cells that may be generated from somatic cells through reprogramming, enabling them to possess embryonic-like properties. Shinya Yamanaka’s group initially derived them in 2006 by reprogramming mouse fibroblasts and human fibroblasts the following year [52,53]. They showed that somatic cells may be converted into pluripotent stem cells by using transcription factors encoded by four specific genes: *c-Myc*, octamer-binding transcription factor 4 (*OCT4*), SRY-box transcription factor-2 (*SOX2*), and Krüppel-like factor 4 (*Klf4*) [52]. The iPS cells are similar to embryonic stem cells (ESC), including morphological similarities: flat colonies with precise edges formed from cells with a high nucleus-to-cytoplasm volume ratio and marked as nucleolus. Similar to ES cells, they have high alkaline phosphatase activity. iPS cells have a gene expression profile characteristic of stem cells. Stage-specific embryonic antigen-3 (SSEA3), stage-specific embryonic antigen-4 (SSEA4), and glycoprotein T-cell receptor α locus (TRA 1-60) and TRA 1-81 are presented on the surface. They may also differentiate into cells from three germ layers and be maintained in a nondifferentiated state for an extended period of time to cultivate cells, known as the self-renewal process [54]. The methylation profile between ES and iPS cells is also similar but differs from somatic cells. Promoters of transcriptionally active genes are characterised by the low methylation level of CpG islands and the presence of the active chromatin marker H3K4me3. Gene promoters responsible for maintaining pluripotency, however, undergo hypomethylation [55]. The determining feature of pluripotency is the reactivation of an inactive X chromosome in female cells and chromatin modifications similar to ESC [56]. 

Using iPSC cells tends to create less of an ethical dilemma than using ESC cells derived from a human embryo inner cell mass. However, the main limitation of the use of iPS cells is linked to the methods employed to obtain them. Most methods for receiving pluripotent stem cells are related to using viral vectors, such as retroviruses and lentiviruses [57]. These vectors integrate randomly into the host cell’s genome, leading to genetic instability or interfering with the appropriate functioning of the integrated genes, and may increase the risk of tumour formation [58]. Recent discoveries, however, have also led to alternative technologies for obtaining iPS cells, such as an adenovirus, plasmid transfusion, or nonintegrating episomal vectors, all of which carry a lower risk of tumour formation [58,59,60]. It has also been established that chemical stimulation can reduce the oncogenic factors supplied to the cells for reprogramming into iPS cells [61]. Different factors may replace the four primary factors present in the Yamanaka cocktail, but the essential factor *OCT4* may not be omitted [62].

It is also worth mentioning the low efficiency of reprogramming. Researchers stress that Yamanaka’s initial research into reprogramming cells into iPS cells had a low success rate regarding their acquisition. Studies have also found that the cell reprogramming efficiency increases in cells where the *TP53* gene is silenced [63]. Therefore, this could provide genetic stability in cells, and some precautions and strict testing strategies are necessary to reduce the risk of cancer development and confirm their safety [64]. It has been suggested that effective cell reprogramming may potentially reduce the Mbd3/NuRD complex (methyl-CpG-binding domain protein 3/Nucleosome Remodeling and Deacetylation). It has been stressed that nuclear reprogramming is inefficient, and the molecular mechanisms for returning epigenetic states during iPSC production are not entirely understood [65]. Researchers have found that lowering the Mbd3 expression of the NuRD subunit improves the reprogramming efficiency and facilitates the creation of pluripotent stem cells, even in the absence of *c-Myc* and *Sox2* [66]. Recent studies have also suggested that Krüppel-associated box domain zinc fingers (KRAB-ZNFs) are responsible for maintaining the pluripotency state by the methylation of genes responsible for pro-differentiation [65].

The above problems lead to another issue commonly related to the grafting of derived tissues and the immunological reactions of recipients [67]. In the case of the autologous transplantation of iPSC-derived tissues, there remains a risk of their rejection [68,69]. It has been suggested that the source of the cells (exhibiting high immunogenicity), late passages of iPSC cultures or by way of reprogramming somatic cells (primarily based on the retroviral vectors), enhances the response to a formed graft [70,71,72]. The reprogramming of cells with low immunogenic potentials, such as mesenchymal cells and cord blood mesenchymal stem cells, could solve these obstacles [69,73]. The recent data also indicated that modifying the expression of MHC-1 class molecules could significantly reduce the rejection of iPSC-based grafts [74]. Another idea is to create the haplotyping of iPSC clones, which results in obtaining universal cell biobanks compatible with the vast majority of potential donors, reducing the cost and time of iPSC derivation for regenerative purposes [75,76,77].

## 4. Comparison of iPSC and MSC

Mesenchymal stromal cells (MSCs) are multipotent cells that can differentiate into osteoblasts, chondrocytes, myocytes, or adipocytes [78,79]. Across the literature, there is another term used for this population, mesenchymal stem cells. The International Society for Cell and Gene Therapy (ISCT^®^) has recently suggested that this term is inappropriate unless the data support their “stem-like” functionality and properties [79]. They are found in the bone marrow (in low amounts: 0.001%), fat tissue (a rich source), umbilical blood and cord, and dental pulp [80,81,82]. The source of these cells should also be indicated and emphasised, because they exhibit different immunomodulatory and paracrine properties [82]. A recent study has shown that bone marrow (BM-MSC), umbilical cord (UC-MSC), and adipose tissue (AT-MSC), all belonging to MSC, had distinct roles in graft-vs-host disease. The AT-MSC- and UC-MSC-derived cells also had higher procoagulant properties, which raises safety concerns [83]. Their application in neuroregenerative purposes, for example, indicated significant differences between BM-MSC, AT-MSC, and UC-MSC secretomes, phenotypes, and growth kinetics, resulting in improved neurite growth in the presence of a conditioned medium from UC- and AT-MSC compared to BM-MSC. However, their protective properties were maintained at a similar level [84]. Some studies have also tested the utility of MSC derived from different sources in cartilage regeneration [81,85,86]. Among them, the best choice for joint regeneration purposes would be the MSC derived from bone marrow [81,85,86]. Despite this, their unique features make them an attractive source of cells for a wide range of medical applications. The limitations of these cells are the invasive way they are procured and the limited ability to provide MSC in significant quantities while maintaining a high quality. It should also be emphasised that their proliferative and differentiation potentials decrease with age and in patients with bone or metabolic diseases.

MSCs are large cells with a round cell nucleus, a marked nucleolus, surrounded by finely dispersed chromatin particles. This lends the nucleus a distinct appearance. The cells have an elongated spindle-like shape. One of the essential criteria for MSCs is that they must exhibit adhesion to plastic surfaces (e.g., the plate on which they grow). On the other hand, iPS cells have a large cell nucleus and a small volume of the cytoplasm. They form sharp-edged, flat, and tightly packed colonies [87,88]. MSC and iPS cells differ significantly in terms of expressed surface markers. MSCs express CD105, CD73, and CD90 but not CD45, CD34, CD14, CD11b, CD79α, CD19, or HLA-DR. In contrast, iPS cells may be characterised by the presence of SSEA-3, SSEA-4, TRA-1-60, TRA-1-81, and TRA-2-49/6E and may also display CD30, CD9, CD50, CD200, and CD90 [89].

Both mesenchymal and induced pluripotent stem cells have promising prospects in disease modelling, tissue engineering, and personalised therapy. Recent reports suggest using MSC in treating diseases such as spinal cord injury, heart disease, Alzheimer’s disease, Parkinson’s disease, type 1 diabetes, burns, strokes, and arthritis [90]. By contrast, iPSCs are among the most promising tools for developing regenerative medicine and are most often reprogrammed from fibroblast cultures, cord blood cells, and peripheral blood mononuclear cells [91]. A significant difference between these cells is that MSC cells can only differentiate into mesenchymal germ layer cells, while iPSCs differentiate into cells from all three layers. This property of iPSC cells creates a high risk of teratoma formation due to potential residual cells in newly formed tissue/organs [92]. Creating optimal, safe, and highly efficient differentiation protocols into desired cells is required. The recent data suggest that using iPSC depletion agents from a cell culture after differentiation could resolve this issue. The usage of inhibitors of stearoyl-CoA desaturase (SCD1) or antibodies that recognise lacto-N-fucopentose I (LNFP I) was found to be helpful in that matter due to their higher specificity toward undifferentiated cells [93,94].

Researchers stress that an increasingly promising way to use stem cells is to combine them with scaffolds. This may provide a greater reparative capacity and survival of the cells. Progress in this field is promising for both regenerative medicine and tissue engineering [90]. Due to foetal-like origins, the derivation of progenitors from iPSC could also rejuvenate them, enabling improved adaptation to the pathologically changed diseased area. It was shown in an example of myocardial repair, where iPSC-derived cardiomyocytes significantly improved the ejection fraction with the observable attenuated remodelling of the myocardium into fibrotic tissue [95].

## 5. iChondrocytes Cell Generation—Protocols

**Figure 1:**
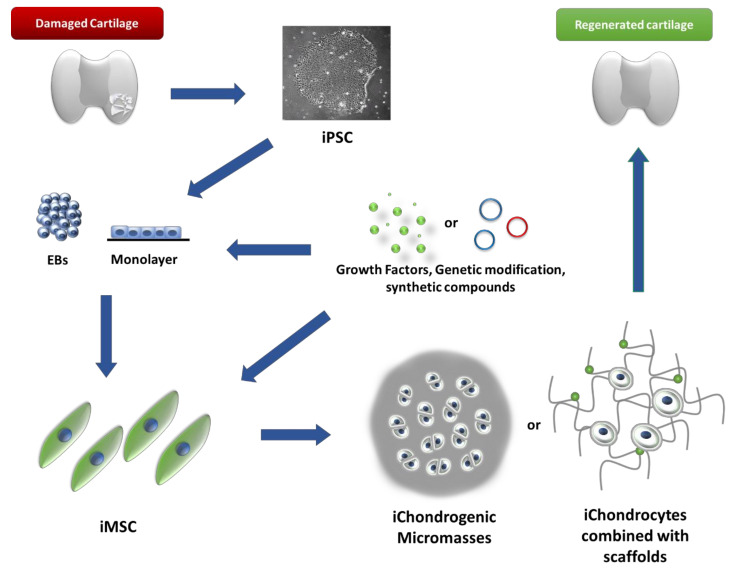
A schematic summarisation of the distinct approaches of iPSC chondrogenic differentiation to repair articular cartilage damage based on the current protocols. To form iPSC’s allogeneic/autologous source, the first step requires the differentiation of iPSC cells into iMSC through the straightforward process or spontaneous iPSC changes into mesenchymal-like stromal cells using an embryoid body step and the additional supplementation of a growth factor to unify this process. The obtained iMSC cells also undergo chondrogenic differentiation using the most common approach through the formation of pellet culture, commonly known as the micromass technique, for some period when the cells mature and produce an appropriate amount of ECM. Other possibilities require the use of biocompatible scaffolds, which also support chondrogenic differentiation and create a ready-to-use patch for large defects in damaged cartilage or a suitable model for skeletal developmental diseases.

The search for effective treatment methods for osteoarthritis has given rise to considerable clinical interest in differentiating iPSC into articular cartilage chondrocytes. As mentioned above, the current techniques are insufficient in the long term and are mainly limited to younger patients with small lesions. Damaged human articular cartilage does not heal itself due to a high content of extracellular matrix and a lack of lymphatic, vascular vessels and neural tissue [96]. A protocol that enables a heightened efficiency in obtaining human chondrocytes that would also be safe for the patient needs to be developed. More importantly, the protocol should provide sufficient cells without changing their phenotype, which would provide high-quality hyaline cartilage after implantation. Most of the basic protocols depend on the induction of mesoderm or mesenchymal cells due to their origin. Many attempts have been made to develop such a protocol using various growth factors, such as fibroblast growth factor (FGF), transforming growth factor-beta (TGF-β), platelet-derived growth factor (PDGF), and modulating different signal pathways with chemical agents [97,98]. The first well-defined protocol in serum-free and chemically defined conditions deserves a special mention. It was developed by the Oldershaw team and was an essential element in future approaches [99]. Briefly, hESC were differentiated step-by-step by the induction of primitive streak/mesendoderm, further mesoderm, and finally, into chondrocyte progenitors for a short period, i.e., 14 days. This protocol required a combination of at least seven growth factors (WNT3A, activin-A, FGF-2, BMP4, Follistatin, GDF-5, and NT-4) and two matrix substrates (fibronectin and gelatin) [99]. These conditions were recently used and modified by different teams of researchers, as discussed below. Another aspect concerns obtaining chondrocytes without exhibiting hypertrophy or terminal maturation over time, leading to ossification and degradation of the repaired cartilage tissue and, consequently, an advance of the disease [100,101]. The following part of this review will focus on differentiation protocols of iPS cells into chondrogenic populations using directed methods or through an embryoid bodies (EBs) step and additional stimuli to improve the formation of cells of a specific germ layer, methods using a cell–scaffold combination, and methods using physical/chemical agents during differentiation (Figure 1 and Table 2).

### 5.1. Differentiation Methods Using Embryoid Bodies (EB)

Embryoid bodies are three-dimensional aggregates of PSC that may be used to obtain three germ layer cells due to their natural properties. The various methods for obtaining EBs were developed, such as a liquid suspension culture in bacterial-grade dishes, a culture in methylcellulose semisolid media, and a culture in hanging drops (HD culture) or even 3D-printed controllable wells of the desired size, which is crucial for this process to maintain a homogenous size and sphericity [125,126,127,128,129,130]. This is a crucial step, because microenvironmental cell culture factors such as oxygenation of the aggregates, access to nutrients, or even a concentration of endogenous growth factors may lead to less efficient differentiation potential into the desired germ layer by the activation of distinct signalling pathways determining their fate [131,132,133]. However, this approach is still popular among scientists, because it is one of the easiest methods of inducing PSC spontaneous differentiation with some control over their fate [125].

Several studies regarding chondrogenic differentiation used that approach with some success (Table 2). The Ko team used that protocol to compare the chondrogenic potential of iPSC with human BMMSC in the regeneration of a knee defect [102]. The EBs formed were dissociated and differentiated into alginate spheres for three weeks in the presence of TGF-β3, known as one of the most pro-chondrogenic growth factors [134]. They have confirmed that iChondrocytes had a higher expression of chondrogenic markers and better regenerative potential than hBMMSC differentiated in the same regimen [102]. Another protocol based on the EB formation step was developed by Zhu et al. [103]. Surprisingly, they repaired a rat’s knee defect in 15 weeks by implanting EB sprouts that had undergone only two weeks’ differentiation in a gelatin monolayer with TGF-β1. COL2 staining, however, was not quite as intense as observed in the surrounding tissue, but at some point, regeneration of the surface occurred in the chemically induced OA (using monosodium iodoacetate) in comparison with the control [103]. An attractive solution was proposed by Li et al., where, for the production of iPSC cells, the peripheral mononuclear cells (PBMC) were reprogrammed using episomal vectors [104]. This allows them to obtain many cells with a less invasive collection method, and besides a low efficiency, a safe source of cells for regenerative medicine purposes was produced [72,104,135]. To improve the chondrogenic potential, after an EB monolayer culture, the cells were also sorted using two positive mesenchymal markers: CD73^+^ and CD105^+^, enhancing the homogeneity of the cell culture. The 3D in vitro and in vivo differentiation indicated significantly increased expression of chondrogenic markers such as COL2, COL9, and AGGRECAN with a high proteoglycans deposition on a comparable level with chondrogenic pellets obtained from MSC [104].

Rim and Nam’s team have published a few interesting papers regarding reprogramming cord blood mononuclear cells (CBMC) for cartilage regeneration [105,136]. Their low immunogenic nature and broad access due to biobanking mean they are likely to be an attractive source of cells for the allogeneic transplantation to treat large cartilage defects. This team developed a chondrogenic protocol based on EB outgrowth cells cultured on gelatin-covered plates and passaged several times, which generated MSC-like cells suitable for differentiation towards chondrocytes in the presence of BMP2 and TGF-β3, a combination that enhances chondrogenic differentiation better than their separate supplementation [137]. However, the addition of several growth factors significantly increases the cost of a cell culture. Therefore, this team of researchers proposed a solution based on the transfection of the EB outgrowth of cells with minicircle vectors encoding BMP2 or TGF-β3 and mixing both populations [105]. This combination resulted in efficient differentiation with a good outcome regarding the repair of osteochondral defects with reduced costs of a cell culture [105].

Another simple protocol was established by the Koyama team, where, using EB outgrowth, they received MSC-like cells and, further, chondrogenically differentiated them into 3D pellets in the presence of TGF-β3. The formed pellets exhibited a weak expression of COL2 and aggrecan production, indicating the early stages of chondrogenesis [106]. They suggested that using additional growth factors, mechanical forces, or a hypoxic cell culture may enhance the maturation and deposition of ECM characteristics for mature hyaline cartilage [138,139]. The authors, however, failed to confirm the in vivo maturation of the derived cells, but their protocol was reproducible in several hiPSC and hESC cell lines, which may be used for disease modelling related to skeletal abnormalities.

The Crafts team proposed a more advanced protocol, which discovered the stable differentiation of iPSC cells using a few events mimicking embryogenesis. Firstly, they induced primitive streak mesoderm (CD56^+^KDR^+^PDGFRα^+^) using the EB approach with exposure to activin A, BMP4, and FGF2; further into a paraxial mesoderm (CD73^+^CD105^+^PDGFRβ^+^) using dorsomorphin; and finally, into chondroprogenitors in the presence of TGF-β3 or BMP4. They established that BMP4 micromasses tend to undergo endochondral ossification by the increased expression of hypertrophic markers rather than with TGF-β3-treated cells. The implanted micromasses indicated a stable chondrogenic phenotype for over 12 weeks after subcutaneous implantation. It was also one of the first protocols of iPSC cultured in serum-free conditions, which implies its preclinical potential [107].

In the same year, Lee’s team presented another suitable protocol based on Oldershaw et al., with a few modifications [99,108]. In the early stages, instead of a monolayer culture, an EB approach with the supplementation of a ROCK inhibitor (Y27632) was used, resulting in the increased viability of cells and the enhanced chondrogenic potential of differentiated cells by a high number of SOX9-positive cells. They exhibited an expression of *WNT9A* and *SOSTCD1* characteristics for ACC from the interzonal part of the histological structure of joint cartilage. After two passages, they matured, which was notified by the presence of a stable and homogenous positive population for SOX9^high^CD44^high^CD140^high^, a marker related to advanced chondrogenesis. They also showed that the 3D culture and in vivo subcutaneous xenotransplantation formed hyaline cartilage without visible pathological changes suggesting tumorigenesis, which support the safety of that protocol.

Boreström’s team, meanwhile, demonstrated that human chondrocytes might be reprogrammed safely with synthetic mRNA and redifferentiated into chondrogenic cells. The first step of the induction of chondrogenesis was unexpectedly related to the culture of a massive cell pellet culture (4 × 10^5^ cells) in the chondrogenic medium in the presence of TGF-β1. The cells were then removed from spheres and expanded for another 14 days in the presence of human serum and, finally, re-pelleted and cultured for another 35 days in the chondrogenic medium from the first step. The limited use of growth factors combined with a lengthy cell culture resulted in spheres expressing high levels of ECM but lower levels of COL2 after differentiation than chondrogenic spheres obtained from donor chondrocytes. However, the lack of in vivo testing did not confirm their utility. Besides the low expression of type II collagen, the spheres expressed comparable type X collagen with donor chondrogenic pellets, which may be explained by the epigenetic memory of OA chondrocytes used in reprogramming.

### 5.2. Direct Methods for Obtaining Chondrocytes from iPSCs

Another part of this review is focused on the monolayer approach, which is more popular than the methods that include the EB step (Table 2). These protocols, however, require the additional supplementation of growth factors and ECM-coating plates, increasing the general costs of stem cell differentiation. However, one great benefit is that this creates a more controllable environment in which to reproduce many cells with a homogenous phenotype than in approaches based on EBs. As mentioned above, their derivation and spontaneous differentiation depend significantly on the cellular mass and culture-well shape. Consequently, a heterogenous population among the derived cells is observed, which requires extra selection steps to obtain a homogenous population. Recent years have brought a tremendous update regarding chondrogenic differentiation protocols with good outcomes, which has created a new platform for anti-OA drug development and studies of chondrogenesis and their pathologies.

The Yang team developed a protocol that modified the Oldershaw et al. approach [110]. They created a simple version of that protocol for drug screening purposes. During differentiation of the keratinocyte-derived iPS cells, they limited the usage of growth factors (NT4 excluded from the protocol), to passage only once in seven days and cultured for another seven days. The plates were also coated with Matrigel alone and optimised for the 96-well format. That simplified and chemically defined protocol has established its suitability for drug testing on the example of two novel peptides, AB235 and NB61, which are new classes of high-affinity TGF-β family ligands [140]. Notwithstanding this, Oldershaw’s and Yang’s protocols are sufficiently fast, but they have some limitations regarding tumorigenesis, as Saito’s group study showed. It was modified to prolong the time for the cell culture for another week (21 days in total) to form the chondrogenic disc, ready to use in the place of cartilage depletion. Sixteen weeks after transplantation, one of the differentiated clones in the implanted knee of the SCID mice developed a teratoma from the remnants of iPSC in the delivered construct. The changes were not observed eight weeks after implantation, suggesting the importance of long-term studies and strict safety procedures regarding tissues formed from iPSC and their genetic stability [64,111].

Yamashita’s team developed a promising protocol of chondrogenic differentiation with colossal potential for producing large cartilage-like particles [112]. The researchers emphasised that the application of BMP2, TGF-β1, and GDF5 factors was essential for the chondrogenic differentiation of hiPSC-derived mesenchymal cells. They also pointed out that the environmental factor and appropriate timing of implantation spheres are crucial to their in vivo maturation. Yamashita’s researchers also developed an efficient way to differentiate hiPSCs into chondrocytes without using scaffolds and embryoid bodies with good repair properties [112]. They also recently indicated the ability of their chondrogenic cells to integrate with the surrounding tissue and itself, which is crucial for maintaining an intact joint surface with the limited progression of OA. They also discovered that FGF-18 was a growth factor secreted by a perichondrium-like membrane, responsible for the ability of obtained cartilage-like particles to integrate [141].

Another way to obtain chondrocytes directly is by using small-molecule compounds. The protocol invented by the Kawata team was the fastest: it took only nine days to obtain chondrogenic-like cells [113]. Their approach was based on using CHIR99021 (6-((2-((4-(2,4-Dichlorophenyl)-5-(4-methyl-1H-imidazole-2-yl)pyrimidine-yl)amino)ethyl)amino)nicotinonitrile) and TTNPB (4-[(E)-2-(5,6,7,8-Tetrahydro-5,5,8,8-tetramethyl-2-naphthalene)-1-propenyl]benzoic acid) [113]. CHIR99021 is a chemical compound that acts as an inhibitor of GSK3 (glycogen synthase kinase 3), responsible for the regulation of the WNT signalling pathway, and mainly causes cell differentiation into the mesoderm [115], TTNPB, meanwhile, otherwise known as arotinoid acid, belongs to the retinoid acid receptor (RAR) antagonist, which plays an important role at the early stages of the formation of limb buds [113,142]. The proposed combination of these two compounds in serum-free conditions resulted in differentiation into chondrogenic-like cells without remnants of pluripotent cells. Their subcutaneous implantation revealed the formation of hyaline cartilage. The 6-month observation of the implanted cells into knee defects in mice also revealed the formation of functional cartilage without the notification of teratoma or tumour formation, supporting the safety of this approach [113]. A similar usage of CHIR99021 was proposed by Kreuser’s team, who revealed that its short administration, no more than 24 h, significantly increases the efficiency of chondrogenic differentiation by improving mesoderm aggregation and condensation, a crucial process during the formation of cartilage nodules [114]. This chemical molecule caused the increased proliferation of cells and the formation of bigger cartilage-like pellets with a dense deposition of ECM compared to the controlled population, leading to a higher number of cells suitable for the coverage of large defects.

A more complex and controllable protocol was proposed recently by the Adkar team, in which they used a cocktail of cytokines and chemical molecules (Table 2), which enabled the highest control of the differentiation process by the regulation of all the developmental stages from pluripotent to sclerotome formation within three days [115]. Besides obtaining a pure population of the early chondrogenic population, in vitro maturation has shown some histological heterogeneity. The authors, therefore, created the reporter cell lines dependent on the expression level of COL2A1. The sorted population exhibited homogenous cartilaginous spheres with stable phenotypes through several passages. Besides the lack of in vivo assays, the in vitro spheres exhibited a high ECM deposition with intense COL2 staining without the presence of hypertrophy or calcifications, which suggests their potency in the regeneration of hyaline cartilage lesions.

Another protocol worth mentioning was proposed by the Guzzo team, which, while not ideal, served as a basic protocol to be adapted by other teams [116,117]. Briefly, they derived the MSC-like cells by spontaneous differentiation via the passaging of iPSC cells in DMEM with FBS and FGF2 until a homogenous population of cells with a spindle-like shape was produced. The morphological changes were intact with their flow cytometric analysis, which revealed the acquiring of BMMSC-like cells with the ability to differentiate into chondrogenic cells in the presence of BMP2 [116]. A similar protocol was proposed by Nejadnik’s team [117]. In their method, iPSC cells were primarily cultivated in the basic medium without FGF on Matrigel-coated plates (for five days) and then transferred onto an uncoated cell culture dish. The differentiated cells exhibited the morphology of MSC cells and characteristic expression of the proteins according to the International Society for Cell Therapy guidelines. Chondrogenically differentiated cells in the 3D pellet with TGF-β3 were then implanted into osteochondral defects of the distal femurs of rats. MRI imaging and a histological analysis revealed a lack of teratoma formation with a good coverage of cartilage depletion. This approach exhibited a slight expression of fibrocartilage and hypertrophic markers, however, which would represent an unacceptable long-term solution, and the authors suggested further improvements of this protocol. One explanation may be related to the basal expression of SOX9 in the derived iMSC, which was shown as a crucial aspect of chondrogenic studies in a number of the Diederich team’s protocols [118,119]. They found that iMSC derived by distinct methods (through EB or spontaneous or BMMSC-conditioned mediums) from the same donor has slightly different global gene expression patterns and affects their functionality compared to parental BMMSC [143]. In the case of chondrogenic differentiation, at their first approach, they observed that a low level of SOX9 expression in MSC-like cells derived from iPSC determines their chondrogenic commitment. Its expression could be partially enhanced by using a combination of TGF-β1 and BMP4, but the in vitro differentiated spheres exhibited the expression of hypertrophic markers. BMP4 induced that effect, so, in their latest protocol, they only used TGF-β1 [119]. Increased reproducibility was also achieved by the usage of cells with a high level of SOX9. They also indicated that cartilage spheres derived from these cells were more resistant to mineralisation than MSC. This led to the conclusion that the derived cells had undergone rejuvenation. A similar observation was noted in the Chang team, which analysed iChondrocytes obtained in comparable conditions in a rabbit model. The researchers demonstrated that the differentiated chondrocytes effectively repaired cartilage defects in vivo and indicated a downregulation of catabolic and proinflammatory cytokines [120]. The possible explanations for this phenomenon were related to the rejuvenation of cells during differentiation and, also, the confirmation of their MSC nature, which is known for its suppressive and protective properties [88].

Another approach was presented by the Aisenbrey team, which was based on the Guzzo approach [121]. They tested the hypothesis of a combination of chemical (growth factors TGF-β3 or BMP2) and physical factors on iMSC differentiation immersed in a nondegradable hydrogel. The hydrogel consisted of multi-arm PEG norbornene macromers, PEG dithiol crosslinkers, and ECM analogues of thiolated chondroitin sulphate. The study established that the highest efficiency was achieved when the culture was performed on hydrogel scaffolds in the presence of TGF-β3 under dynamic mechanical stimulation. The method developed was effective in both promoting chondrogenesis and simultaneously reducing hypertrophy. The researchers emphasised that additional research is needed on a degradable cartilage-like hydrogel to gain significant clinical value [121]. Another recently developed quick method was based on bioceramic products that contained lithium: Li_2_Ca_4_Si_4_O_13_ (L2C4S4) [122]. Lithium itself is a cheap WNT agonist that displays pro-chondrogenic properties during the differentiation of MSC [144,145]. The culture of iPSC in L2C4S4 (3.125–12.5 mg/mL) in the commercially available serum-free chondrogenic medium for 14 days caused successful chondrogenic differentiation with a high expression of chondrogenic markers, i.e., COL2, SOX9, aggrecan, and a low expression of hypertrophic markers (COL10 and MMP13). The extracts of the lithium compounds were shown to have promoted the formation of well-formed spheres, suggesting that L2C4S4 could help maintain the morphology of iPSC-derived chondrocyte spheres. This study confirmed that the action of independent Li^+^ ions at different concentrations could also support the differentiation of iPSCs in chondrocytes and further prevent their hypertrophy. In conclusion, the scaffolds presented helped maintain the appropriate shape and volume of iPSC-derived chondrocyte spheres and supported the rapid conversion of iPSCs into mature chondrocytes [122]. There is, however, a lack of in vivo assays to confirm their ability to repair defects.

One the interesting ideas of differentiation iPSC towards chondrogenic-like populations of cells is the, being cocultured with chondrocytes via culture inserts, as presented by Qu et al. [123]. The advantage of cocultured cells in the well inserts is that the autocrine and paracrine factors secreted by one cell may readily be used and limit the labware’s usage to prepare the conditioned medium itself [146]. The cells exhibited typical chondrogenic markers and a lack of pluripotent cells. After four weeks of pellet culture, they formed homogenous cartilaginous spheres that clearly expressed COL2 and ECM deposition, especially of cells cultured on hyaluronan rather than on gelatin-coated plates. Their monolayer culture through several passages also revealed a similar behaviour as a culture of naïve chondrocytes, which was observed by a decrease in the expression of COL2 with the simultaneous elevation of COL1, which is a common problem in monolayer cultures of primary chondrocytes. Along with these promising results, this study had some drawbacks. Firstly, the coculture of human iPSC cells was conducted in the presence of bovine primary chondrocytes, which could increase the risk of a possible transfer of zoonotic diseases and exclude them from clinical use. Another was related to the lack of confirmation of the expression of hypertrophic markers, but in return, they tested the ability of the cells obtained to differentiate into adipogenic or osteogenic cell populations. Mineralisation has not occurred in iPSC-derived cocultured chondrocytes, unlike MSC cells exposed to these conditions, suggesting their reduced ability to undergo hypertrophy [123].

Using human cells in a similar approach, the Wei team has differentiated reprogrammed OA chondrocytes into chondrogenic-like cells [124]. In this study, to limit the supplementation of growth factors, the authors decided to transduce iPSCs with transforming growth factor-β1. To maintain their stable phenotype, they also used alginate-coated inserts, a natural polymer with documented pro-chondrogenic properties [147]. The combination of these factors resulted in the increased expression of hyaline cartilage marker COL2 in in vitro and in vivo models compared to non-transfected or iPSC cells cultured without improvement. This study gave rise to an intriguing observation: VEGF expression persisted in the differentiating iPSC alone and transduced with TGF-β1, which could be related to the epigenetic memory of OA chondrocytes, since this marker is connected to advanced stages of the differentiation of chondrocytes. However, its expression was inhibited in the presence of a coculture system. The authors suggested that the phenomenon may be linked to the presence of endogenous inhibitors of angiogenesis secreted by cocultured chondrocytes [124,148].

## 6. The Future—Clinical Use

Over a decade, several protocols with satisfactory efficiency, including in vivo characterisation, were established without further application in the clinic. Clinical trials using iPSC in regenerating damaged tissues have been recently used in an example of age-related macular degeneration (AMD) restoration or neural damage [149,150]. In the case of musculoskeletal diseases, there is still a lack of their common usage. One reason is the lack of a well-established, unified differentiation protocol for their derivation with GMP standards [151]. Some protocols still rely on animal components such as foetal bovine serum and feeder cells. This could result in a variation of culture conditions and a risk of the transmission of zoonotic diseases, as well as ethical issues [152,153]. Moreover, the usage of serum in a chondrogenic cell culture has a negative impact on the differentiation process [154,155]. Another problem relates to the source of the cell for chondrogenic differentiation. Several studies have indicated that using a cell from the same donor could also influence the efficiency of chondrogenic differentiation, and the derivation of iMSC cells also revealed that they could not be equally compared to those derived from bone marrow [143,156].

A few issues were not entirely solved in the proposed protocols. One of them is related to teratoma formation. A few protocols have shown that they did not observe any formation of teratoma in the in vivo models, and the expression of pluripotent markers was not present in the neocartilage or iChondrocytes, but in Saito et al.’s study, despite that fact, some of the cells remained, and in one tested subject, they observed the formation of a tumour [111]. However, to improve that outcome, the usage of PluriSln, a synthetic inhibitor of SCD1, could deplete the remnant PSC cells. It was already tested during the chondrogenic differentiation of hESC with a good outcome and lack of cytotoxic effect to the chondroprogenitors [93,157]. Another possibility, published recently, was related to the direct usage of antibodies, highly selective for iPSC, which enabled eradicating them without the affection of mature chondrocytes [94]. There are some common doubts related to the usage of iPSC cells in regenerative medicine due to their potential immune responses [69,70]. Cartilage itself is a tissue with low immunogenic potential due to the lack of vascularity, a high amount of dense ECM disabling the penetration of the immune cells. Moreover, the usage of scaffolds in the implantation site or large cartilage particles with dense ECM could limit the rejection of newly formed tissue [158,159]. Using iPSC technology, the immune response could be omitted if the cells are derived from the same patient, but due to the vast costs of reprogramming and set of assays confirming their safety, the usage of universal haplotyping biobanks could resolve that issue, decreasing the cost of the procedure [160,161].

A few well-described protocols with rather good outcomes have been developed. However, Yamashita’s group established one of the most promising protocols with the best combination of growth factors (TGF-β1, GDF-5, and BMP2). Their findings have been supported by several papers describing their approach as safe and reproducible, with immunogenicity similar to native chondrocytes; they have long-lasting observations without the notification of teratoma formation and good regeneration properties in large animals with mid-sized defects (mini-pig model), and importantly, their construct is scaffoldless [112,159,162,163]. These findings suggest that distinct routes for obtaining iChondrocytes for regenerative purposes are possible. The variability of the protocols with quite good outcomes will enable their adaptation and everyday use, dependent on the economic status of orthopaedic centres. The new source of cells could replace the autologous chondrocytes in the standard techniques, such as MACI, where their combination with the approved by the FDA commonly used collagen matrix could decrease the time to make this solution for common use [164]. This could be a game-changer, because the chondrogenic cells obtained from iPSC also seem to be more resistant to hypertrophy due to their rejuvenation, and the procedure could be reduced to one surgery, diminishing the costs and the patient’s discomfort.

## 7. Disease Modelling from Chondrogenic iPSC

Despite the lack of the use of existing differentiation protocols in the clinic, their establishment could be used in another practical way: the design of disease models regarding limb development. Developmental biology and the usage of iPSC in this well-known process could provide an insight into pathological conditions, leading to better diagnostics and the development of drug screening platforms for those diseases. One of the problems with modelling the diseases is obtaining sufficient tissue for analysis. This involves invasive procedures and general discomfort for the patient and a reduced quality of life for the patients, especially children and young adolescents [165,166,167]. Obtaining PBMC or dermal fibroblasts is less controversial in testing and formation modelling diseases, especially involving limb development. Patient-specific derived tissues enable matching the appropriate treatment regimen and doses of potential drugs. It may also eliminate the usage of animal testing, since their physiology and response to the treatment is unlike that in human physiology [168]. The specimens of skeletal abnormalities are difficult to collect because of cartilage’s limited healing ability and may be invasive for the tested patient. Table 3 summarises the distinct models of diseases and iPSC derived from patients with specific mutations related to chondrogenesis and the formation of long bones in endochondral ossification.

Chondrodysplasias are a group of severe diseases caused by disorganised development of the limbs and chondrogenesis itself. One of the most common mutations in the fibroblast growth factor 3 (*FGFR3)* gene, a negative regulator of bone development, is abnormally activated in these diseases. This leads to inappropriate skeletal formation via disorganisation of the endochondral ossification process [180]. The Kimura and Yamashita team created a model of achondroplasia, hypochondroplasia, and thanatophoric dysplasia based on their differentiation protocols [112,163,169]. They indicated that the use of statins might be helpful in the correction of bone growth in mice and repair the loss of the function of chondrocytes during their differentiation [163]. Similar observations were noted using the FGFR3 inhibitor, NVP-BGJ389, where xenotransplanted iPSC-derived chondrocytes from patients with FGFR3 mutations indicated a restoration of their function [169]. Interestingly, the model of this disease could also be formed artificially. Horie’s team used the CRISPR/Cas9 method to generate a model of achondroplasia (p.G380R). In vitro differentiation of these cells revealed abnormalities via the downregulation of the Indian hedgehog signalling molecule (*IHH*). This confirmed the correctly obtained model, since increased IHH is responsible for the maturation and pre-hypertrophy of chondrocytes [181]. 

Another group of disorders related to chondrogenesis that was studied using iPSC modelling is connected with the accumulation of proteins in the intracellular compartments of chondrocytes, leading to the activation of the unfolded protein response (UPR) [171,172,182]. Among them, we may distinguish multiple epiphyseal dysplasia (MED) and metaphyseal chondrodysplasia type Schmid (MCDS), which are related to the widening and irregularity of growth plates, an increased risk of developing early osteoarthritis of the knee and hip [171,183,184]. These diseases are related to the occurrence of mutations in type 10 collagen (*COL10A1*), a marker of hypertrophic chondrocytes, and matrilin-3 (*MATN3)*, the protein responsible for terminal chondrogenic differentiation and homeostasis of the cartilage [171,181,185,186]. A recently published model of this disease by the Pretemer team revealed that the endoplasmic reticulum was significantly enlarged after the chondrogenic differentiation of COL10A1 and MATN3 mutants [171]. Histological staining also revealed the increased deposition of COL10 and matrilin-3 intracellularly in several mutants, a state partially reversed by using trimethylamine N-oxide (TMAO), an agent acting as a chemical chaperone [171]. This compound was also studied in other diseases linked to the retention of type II collagen, a protein highly expressed in hyaline cartilage chondrocytes, which enables the stiffness and bearing of the mechanical loading of cartilage [172]. In general, those type II collagenopathies exhibit a lack of genotype–phenotype correlation and primarily present as abnormalities of ocular, otolaryngological, and skeletal systems with either a mild or severe bout of disease [187,188]. Interesting studies regarding that type of disease on the example of iPSC cell lines derived from patients with achondrogenesis type II (ACGII), hypochondrogenesis (HCG), and spondyloepiphyseal dysplasia (SPD) have shown an increased deposition of COL2 intracellularly and increased apoptosis of the affected cells. The use of TMAO, as in the above study, partially reduced the ER stress, enabled the secretion of type II collagen in the extracellular environment, and increased viability of the mutated cells [172]. Another rare disease related to the accumulation of mutated proteins in the intracellular compartments of chondrocytes with severe skeletal deformation outcomes is Familial Osteochondritis Dissecans (FOCD) [173]. The pathobiology of FOCD is linked with mutations in aggrecan (ACAN), a crucial component of articular cartilage proteoglycans, enabling the formation of dense ECM that protects chondrocytes and improves the binding of water molecules to maintain the biomechanical function of joints [189]. The analysis of chondrogenic pellets and nodules derived through the differentiation of iPSC from FOCD patients revealed a reduced presence of ACAN and ECM production compared to in healthy control. In addition, the increased deposits of ACAN were allocated in the ER, causing a severe stress response in those cells. The mass spectrometric analysis of the components of ECM revealed the increased expression of peptides, small leucine-rich proteoglycans (SLRPs), linked to OA and mineralisation during osteogenesis [173,190]. The chondrogenic differentiation of bone marrow MSC derived from the patients diagnosed with FOCD compared to iPSC-derived cells also revealed similarities that confirmed their usefulness as a model of that disease and potential platform for studying potential therapeutic approaches.

The Yokoyama team developed another successfully developed disease model. They created a model of neonatal-onset multisystem inflammatory disease (NOMID), a rare disease caused by a mutation in the NLR family pyrin domain-containing 3 (NLRP3) gene encoding protein cryopyrin [174]. That mutation caused a pathological abnormal systemic inflammation response via the increased formation of multiprotein complexes called inflammasomes. Their activation leads to the increased release of IL-1β, a widely known cytokine responsible for increased catabolic reactions in cartilage [191,192]. One of the manifestations of the disease is related to the enlarged epiphyseal and disorganised structure of isogenic groups of cartilage [193]. The chondrogenic in vitro differentiation of mutant iPSC cells resulted in large cartilaginous masses with increased ECM/GAG production. The NOMID-derived chondrocytes also matured in vivo and exhibited a disorganised endochondral ossification process and increased mass. Their study explained that the enlarged cartilaginous masses were related to the co-upregulation of SOX9, the master transcription factor of chondrocytes, via the cAMP/PKA/CREB signalling pathway in the chondrogenically differentiated, mutated iPSC clones [174].

Among models of skeletal developmental disorders, metatropic dysplasia was established by the Saitta team using cells from a patient carrying a lethal mutation (C1812>G) of the transient receptor potential cation channel, subfamily V, member 4 (*TRPV4*) [175]. This defect is responsible for the dysregulation of Ca^2+^ homeostasis of chondrogenic or neural cells [194]. This type of disease correlates with metaphyseal enlargement, dwarfism, cervical instability, and severe kyphoscoliosis [195,196]. The in vitro chondrogenic differentiation of mutated iPSC clones revealed the reduced production of COL2 with a parallel increased production of COL1 in comparison with healthy iPSC. The gene expression analysis also showed that COL10A1 and RUNX2, markers characteristic of the late stages of chondrogenesis, were downregulated compared to the control. This explains why the hypertrophic zone of articular cartilage was reduced and chondrogenic maturation was weakened [175]. 

The Matsumoto team made another interesting observation. They established the iPSC model from a patient with fibrodysplasia ossificans progressiva (FOP) [176]. This rare genetic disease is characterised by replacing muscle and connective tissue with bone due to severe damage, invasive procedures, or infectious diseases [197]. One characteristic trait of the disease is the malformation of the great toes. The mutations in the activin receptor type I (*ACVR1)* are responsible for the occurrence of the disease, which is related to its binding ability of BMP proteins known for their osteoinductive properties [198,199]. In this model, the differentiated cells exhibited a higher deposition of ECM and an expression of chondrogenic markers (SOX9, COL2A1, and ACAN) than with the control iPSC cells (genetically corrected FOP-iPSC). The comparative gene expression profile of the mutated and corrected iPSC cells enabled researchers to identify MMP1 and PAI1 as the proteins responsible for enhanced chondrogenesis and its partial activation via the SMAD signalling pathway. The chemical inhibitors (GM6001 and tiplaxitin) partially reduced the production of GAG and the area of chondrogenic micromasses cultured 2D [176].

In addition to several types of skeletal dysplasia, a number of OA models have also been created, such as hand OA, early-onset finger OA, and chemically induced OA (stimulation of a formed osteochondral plug in a dual-flow bioreactor with IL-1β) [177,178,179]. The introduction mentioned that OA is a complex disease with several environmental and genetic background factors [12]. Both the Rims and Castro-Viñuelas teams’ studies yielded similar data, showing the less chondrogenic potential of iPSC from OA patients than in healthy donors. It was assessed by the reduced expression of hyaline cartilage markers deposition of the ECM with a parallel increased expression of hypertrophic markers related to the late stages of chondrogenesis [177,178]. Lin et al. carried out an interesting study, in which they created a construct that consisted of mesenchymal progenitor cells derived from iPSC cells suspended in methacrylate gelatin, which was used to create an osteochondral plug in the dual-flow bioreactor [179]. The bottom of the construct was exposed to an osteogenic cell culture medium, and the top was rinsed in a chondrogenic medium. This allowed the creation of the first model imitating a full-thickness osteochondral plug for testing drugs. They confirmed the utility of that model by inducing OA using IL-1β, which indicated a reduced expression of chondrogenic markers (ACAN and COL2) and increased cartilage-degrading proteins (MMPs and ADMTS). The use of Celecoxib, a potent disease-modifying osteoarthritis drug, showed its chondroprotective properties by reducing the catabolic and proinflammatory induced response [179].

It is worth mentioning that the recent data indicated the derivation of new iPSC cell lines from patients with cleidocranial dysplasia (deletion of exon 3 of *RUNX2*) and acromesomelic dysplasia (*PRKG2,* frameshift insertion). Neither has the effect of those disorders yet to be elucidated on in vitro or in vivo chondrogenesis [200,201].

## 8. Conclusions

The recently obtained breadth of knowledge concerning the chondrogenesis process and the establishment of several well-defined differentiation protocols with good in vivo outcomes could yield a strong base for forming clinical trials in that area soon. For now, these protocols enable the discovery of the bias of skeletal development pathologies and establish practical disease models for the screening of drugs and studies of mechanisms explaining the biology of those diseases without the necessity of an extensive collection of materials from patients.

## Figures and Tables

**Table 1:** A summary of the most used techniques for the repair of damaged cartilage.

Technique Repair	Advantages	Disadvantages	Ref
MACI	+ cells seeded on scaffolds + lack of leakage from periosteal patch + speedy recovery + less invasive + improved mobility	- fibrocartilage formation - expensive method of treatment - requires two surgeries - limited to small lesions	[23,24]
ACI	+ speedy recovery + less invasive + improved mobility + reduced pain + increased joint motion	- requires two surgeries - limited to active young people - chondrocyte dedifferentiation - leakage of cells from periosteal patch - damage to healthy tissue - dedifferentiation of chondrocytes - fibrocartilage formation - limited to small lesions	[22,25,26]
Microfracture	+ one-step surgery + low cost + simple, non-invasive procedure.	- fibrocartilage is formed - age limit - the necessity of subsequent surgeries in the long term	[4,27,28,29]
Mosaicplasty and OAT	+ coverage of deep cartilage lesions, + exact full-thickness composition of joint tissue is used, + deep and medium-deep lesions may be treated, + speedy recovery and excellent short-term results, + mid-sized damage (over 3 cm^2^) may be repaired	- obtaining healthy tissue for grafts, - severe damage of tissue - the morbidity of the site of collection - problems with integration in the donor site - the loosening of the cylinder in the long-term - fibrocartilage formation between cylinders	[23,30,31,32,33,34]
Joint Arthroplasty	+ reduction in joint pain and stiffness + increased performance of daily activities (ADLs) + increased joint mobility + extensive damage to tissue may be treated + in terms of joint resurfacing, physical activities can be maintained	- invasive procedure, - high risk of infection - high risk of post-surgical complications - expensive cost of prosthetics, - less stable joint due to a lack of native structure of joint - revisions surgery - an adverse reaction to metal debris (ARMD)	[35,36,37,38]

ACI: autologous chondrocyte implantation; ADL: daily activities; ARMD: an adverse reaction to metal debris; MACI: matrix-induced autologous chondrocyte implantation; OAT: osteochondral autograft transplant.

**Table 2:** The protocols of chondrogenic differentiation of iPSC cells with the best outcomes among the studied variants.

System of Culture	Differentiation Factor	Duration	In Vivo Confirmation	Serum-Free	Ref.
EB→3D PELLET	Stage 1: Mesenchymal differentiation through EB, ATRA (10^−7^ M) Stage 2: Chondrogenesis: alginate (2%), TGF-β3 (10 ng mL^−1^)	33 days	Yes (osteochondral defect of the knee, rat) Outcome: 12 weeks after implantation, the macroscopic (ICRS) and histologic evaluation revealed better regenerative properties of iPSC-derived chondrocytes compared to the control group; Lack of teratoma formation was observed	No	Ko et al., 2014 [102]
EB→MONOLAYER	Stage 1: Mesenchymal induction through EB formation Stage 2: Chondrogenesis: TGF-β1 (10 ng mL^−1^)	14 days	Yes (osteochondral defect of the knee, rat) Outcome: 15 weeks after implantation, the Micro-CT images and macroscopical evaluation has shown the repair of the damaged area on the surface, but the subchondral trabecular bone was not recovered; Histological analysis confirmed neocartilage formation and expression of COL2 and GAGs; Lack of teratoma development was notified	No	Zhu et al., 2016 [103]
EB→MONOLAYER → 3D PELLET	Stage 1: Collection of EB outgrowth Stage 2: Chondrogenic differentiation TGF-β1 (10 ng mL^−1^)	48 days	Yes (kidney capsule, mice) Outcome: 6 weeks after implantation, the histological analysis revealed the GAGs deposition and presence of COL2 and COL10; Lack of teratoma was observed	No	Li et al., 2016 [104]
EB→MONOLAYER→3D PELLET	Stage 1: EB culture TGF-β1 (2 ng mL^−1^) Stage 2: Outgrowth of EB transfected with *BMP-2* or *TGF-β3* Stage 3: *BMP2* or *TGF-β3* transfected outgrowth EB mixed equally and cultured 3D	47 days	Yes (osteochondral defect of the knee, rat) Outcome: 8 weeks after implantation of chondrogenic pellets, the damaged area was restored with the tissue containing a high level of GAG, COL2 and low amount of COL1 or COL10; Lack of teratoma formation was notified.	No	Rim et al., 2020 [105]
EB→MONOLAYER→3D PELLET	Stage 1: Collection of EB outgrowth Stage 2: Chondrogenic differentiation TGF-β3 (10 ng mL^−1^)	42 days	No	No	Koyama et al., 2013 [106]
EB→MONOLAYER→3D PELLET	Stage 1: Activin A (2 ng mL^−1^); BMP-4 (3 ng mL^−1^); FGF2 (5 ng mL^−1^), CHIR99021 (1 µM) Stage 2: Dorsomorphin (4 µM); FGF2 (10 ng mL^−1^), SB431542 (5.4 µM) Stage 3: TGF-β3 (10 ng mL^−1^)	25 days	Yes (subcutaneously, mice) Outcome: 12 weeks after implantation, the histological analysis of formed pellets has shown high expression of COL2, GAGs and low content of COL1; There was a lack of signs of mineralisation or teratoma formation	Yes	Craft et al., 2015 [107]
EB→MONOLAYER→3D PELLET	Stage 1: Mesoderm induction during EB formation: WNT3A (25 ng mL^−1^), Activin A (50, 25,10 ng mL^−1^), FGF2 (50 ng mL^−1^) and BMP4 (40 ng mL^−1^) Stage 2: FGF2 (50 ng mL^−1^), BMP4 (40, 20 ng mL^−1^), Follistatin (100 ng mL^−1^), NT4 (2 ng mL^−1^) and GDF5 (20, 40 ng mL^−1^) Stage 3: IGF (N.A.), FGF2 (N.A)	42 days	Yes (subcutaneously, mice) Outcome: 4 weeks after implantation, the harvested pellet exhibited formation of cartilage containing a high amount of COL2 and ECM, without COL10 expression; Lack of development of teratoma	No	Lee et al., 2015 [108]
3D PELLET→MONOLAYER→3D PELLET	Stage 1: Predifferentiation TGF-β1 (10 ng mL^−1^) Stage 2: Expansion (Human Serum) Stage 3: maturation TGF-β1 (10 ng mL^−1^)	70 days	No	No	Boreström et al., 2014 [109]
MONOLAYER	Stage 1: WNT3A (25 ng mL^−1^); Activin A (50,25,10 ng mL^−1^); FGF2 (20 ng mL^−1^); BMP4 (40 ng mL^−1^); Follistatin (100 ng mL^−1^) Stage 2: BMP4 (40 ng mL^−1^); FGF2 (20 ng mL^−1^); GDF-5 (40 ng mL^−1^)	14 days	No	Yes	Yang et al., 2012 [110]
MONOLAYER→3D DISK	Stage 1: WNT3A (25 ng mL^−1^), Activin A (50, 25, 10 ng mL^−1^), FGF2 (20 ng mL^−1^), BMP4 (40 ng mL^−1^) Stage 2: FGF2 (20 ng mL^−1^), BMP4 (40 ng mL^−1^), Follistatin (100 ng mL^−1^), NT4 (2 ng mL^−1^) Stage 3: NT4 (2 ng mL^−1^), FGF2 (20 ng mL^−1^), GDF-5 (20, 40 ng mL^−1^), BMP4 (20 ng mL^−1^) Stage 4: NT4 (2 ng mL^−1^), FGF2 (20 ng mL^−1^), GDF-5 (40 ng mL^−1^)	21 days	Yes (osteochondral defect, mice) Outcome: 8 and 16 weeks after transplantation, the newly formed cartilage tissue was observed with dense ECM; one of the tested subjects has developed the teratoma	Yes	Saito et al., 2015 [111]
MONOLAYER→3D PELLET	Stage 1: Mesoendodermal induction: WNT3A (10 ng mL^−1^), Activin A (10 ng mL^−1^) Stage 2: Chondrogenesis: BMP2 (10 ng mL^−1^), TGF-β1 (10 ng mL^−1^), GDF5 (10 ng mL^−1^)	42 days	Yes (subcutaneously, mice; Osteochondral defect, rat; Osteochondral defect, mini pig) Outcome: In mice, after 12 weeks, the implanted spheres exhibited microscopical morphology of hyaline cartilage with high ECM deposition and high COL2 expression; After 12 months, the implanted pellets underwent hypertrophy and maintained epiphyseal morphology; In rats, after 4 weeks, the osteochondral defect was restored with cartilage tissue expressing high COL2 and high GAG content with good integration with surrounding tissue; In mini-pigs, after 4 weeks the damaged cartilage was restored with visible integration with host tissue; Lack of teratoma formation in tested subjects	No	Yamashita et al., 2015 [112]
MONOLAYER	Stage 1: Mesoendoderm CHIR99021 (10 µM ) and TTNPB (100 nM) Stage 2: Chondrogenesis TTNPB (100 nM)	9 days	Yes (subcutaneously, mice; osteochondral defect of the knee, mice) Outcome: After 8 weeks, histological analysis of subcutaneously implanted disc revealed high ECM and COL2 deposition without COL10 expression; After 6 months, histological analysis of the knee revealed the formation of hyaline cartilage enriched in COL2, ECM, and lack of COL10; Lack of teratoma was notified; The histological score was better than in the treated group.	Yes	Kawakata et al., 2019 [113]
MONOLAYER→3D PELLET	Stage 1: CHIR99021 (5 µM), FGF2 (4 ng mL^−1^) Stage 2: TGF- β1 (10 ng mL^−1^)	56 days	No	No	Kreuser et al., 2020 [114]
MONOLAYER→3D PELLET	Stage 1: Mesodermal lineage induction: Activin A (30 ng mL^−1^), SB505124 (2 µM), CHIR99021 (4, 3 µM) , FGF2 (20 ng mL^−1^), C59 (1 µM), Dorsomorphin (4 µM) , PD173074 (500 nM), Purmorphamine (1 µM) Stage 2: Prechondrogenesis: BMP4 (20 ng mL^−1^) Stage 3: Chondrogenesis: TGF-β3 (10 ng mL^−1^)	43 days	No	Yes	Adkar et al., 2019 [115]
MONOLAYER→3D PELLET	Stage 1: Mesenchymal induction through spontaneous differentiation, FGF2 (5 ng mL^−1^) Stage 2: Chondrogenic differentiation BMP2 (100 ng mL^−1^)	50 days	No	No	Guzzo et al., 2013 [116]
MONOLAYER→3D PELLET	Stage 1: MSC induction spontaneous differentiation Stage 2: Chondrogenic differentiation TGF-β3 (10 ng mL^−1^)	56 days	Yes (Osteochondral defect of the knee, rat) Outcome: After 6 weeks, the implanted spheres have shown good integration with the surrounding tissue, increased GAG and COL2 expression; The ICRS score was the highest in the iPS-derived chondrocytes	No	Nejadnik et al., 2015 [117]
MONOLAYER→3D PELLET	Stage 1: mesenchymal progenitor induction FGF (4 ng mL^−1^) Stage 2: Chondrogenesis TGF-β1 (10 ng mL^−1^) and BMP4 (100 ng mL^−1^)	70 days	No	No	Diederichs et al., 2016 [118]
MONOLAYER→3D PELLET	Stage 1: mesenchymal differentiation: FGF (4 ng mL^−1^) Stage 2: Chondrogenesis: TGF-β1 (10 ng mL^−1^)	42 days	No	No	Diederichs et al., 2019 [119]
MONOLAYER	Stage 1: Spontaneous differentiation into MSC in low glucose DMEM Stage 2: Chondrogenesis TGF-β1 (10 ng mL^−1^)	49 days	Yes (osteochondral defect of the knee, rabbit) Outcome: After 12 weeks, histological analysis revealed better microscopical integration, increased ICRS score, higher aggrecan content and better regeneration than the control group; lack of teratoma formation	No	Chang et al., 2020 [120]
MONOLAYER→3D HYDROGELS	Stage 1: Mesenchymal induction through spontaneous differentiation, FGF2 (5 ng mL^−1^) Stage 2: Chondrogenic differentiation in the presence of TGF-β3 (10 ng mL^−1^) and mechanical loading	50 days	No	No	Aisenbrey et al., 2019 [121]
3D PELLET	Extracts from Li2C4S4 bioceramic (12.5, 6.25, 3.125 mg mL^−1^) in commercial MCDM medium	14 days	No	Yes	Hu et al., 2020 [122]
MONOLAYER(HYALURONAN)	Co-culture with primary bovine chondrocytes, TGF-β3 (10 ng mL^−1^)	21 days	No	No	Qu et al., 2013 [123]
MONOLAYER (SCAFFOLDS)	iPSC transduced with *TGF-β1* and co-culture with chondrocytes	14 days	Yes (Subcutaneously, mice) Outcome: After 6 weeks, histological analysis confirmed the highest expression of COL2 and formation of lacuna in *TGF-β1*/Alginate compared to other variants; lack of teratoma formation	No	Wei et al., 2012 [124]

ATRA: all-trans retinoic acid; BMP2: Bone Morphogenetic Protein 2; BMP4: Bone Morphogenetic Protein 4; C59: 2-(4-(2-methylpyridin-4-yl)phenyl)-N-(4-(pyridin-3-yl)phenyl)acetamide; CHIR99021: 6-2-4-(2,4-dichlorophenyl)-5-(5-methyl-1H-imidazol-2-yl)-2-pyrimidinylaminoethylamino-3-pyridinecarbonitrile; COL2: type II collagen; COL10: type X collagen; DMEM: Dulbecco’s Modified Eagle’s Medium; EB: embryoid bodies; ECM: extracellular matrix; FGF2: Fibroblasts Growth Factor 2; GAG: glycosaminoglycans; GDF5: Growth Differentiation Factor 5; ICRS: International Cartilage Repair Society; MCDM: mesenchymal stem cell chondrogenic medium; MSC: mesenchymal stromal cell; NT4: Neurotrophin-4; PD173074: N-2-4-(diethylamino)butylamino-6-(3,5-dimethoxyphenyl)pyrido2,3-dpyrimidin-7-yl-N′-(1,1-dimethylethyl)urea; SB505124: 2-(5-benzo1,3dioxol-5-yl-2-*tert*-butyl-3H-imidazol-4-yl)-6-methylpyridine hydrochloride hydrate; TGF-β1: transforming growth factor β1; TGF-β3: transforming growth factor β3; TTNPB: 4-[(E)-2-(5,6,7,8-tetrahydro-5,5,8,8-tetramethyl-2-naphthalenyl)-1-propenyl]benzoic acid; WNT3A: Wingless/Int1 family member 3a.

**Table 3:** The skeletal developmental diseases that are used as models with iPSC formation.

Disease	Factor/Mutation	Results	Ref
Achondroplasia (ACH), Hypochondroplasia (HCH) and Thanatophoric dysplasia (TD)	ACH: *FGFR3* c.1130G>A (p.G380R) HCH: *FGFR3* c.1620C>G (p.N540K) TD1: *FGFR3* c.742C>T (p.R248C) TD2: *FGFR3* c.746C>G (p.S249C)	The FGFR inhibitor (NVP-BGJ398) has corrected the failure of growth plate formation during chondrogenic differentiation.	Kimura et al., 2018 [169]
Achondroplasia (ACH) and Thanatophoric dysplasia (TD)	ACH: *FGFR3* c.1130G>A (p.G380R) TD1: *FGFR3* c.742C>T (p.R248C)	The usage of statins during the chondrogenic differentiation of patient-derived iPSC has enabled the formation of cartilage micromasses and caused elongation of the bones in transgenic mice	Yamashita et al., 2014 [163]
Achondroplasia	ACH: *FGFR3* c.1138G>A (p.G380R)	Genetically modified cell line using CRISPR/Cas9. Downregulation of *IHH* in mutated cells leads to impaired maturation of chondrogenic cells	Horie et al., 2017 [170]
Multiple epiphyseal dysplasia (MED) and metaphyseal chondrodysplasia type Schmid (MCDS)	MED: *MATN3* c.359C>T (p.T120M) *MATN3* c.659T>C (p.V220A) *MATN3* c.626G>C (p.R209P) MCDS1: *COL10A1* c.1841_1841delT (p.L614Rfs*8)) MCDS2: *COL10A1* c.53G>A (p.G18E) *COL10A1* c.1798T>C (p.S600P)	Intracellular retention of MATN3 and COL10 in mutated cells leads to UPR response, and its activation-dependent on the mutations. The in vitro model for drug testing and development was formed	Pretemer et al., 2021 [171]
Type II collagenopathy: -Achondrogenesis type II (ACGII) -Hypochondrogenesis (HCG) -Spondyloepiphyseal dysplasia (SPD)	ACGII-1: T>C, exon 41-intron 40, exon skipping ACGII-2: *COL2A1* c.3545G>A (p.G1182A) HCG-1: *COL2A1* c.1348G>C (p.G450R) SPD-1: *COL2A1 c.*4337dupG	Intracellular accumulation of COL2 decreased viability of obtained iChondrocytes. It increased ER-stress signalling due to a disturbed regulation of protein folding. The application of chemical chaperones could partially improve the secretion of COL2.	Okada et al., 2015 [172]
Familial Osteochondritis Dissecans (FOCD)	*ACAN* c.6907G>A (p.V2303M)	Increased accumulation of aggrecan in cells caused ER stress. Abnormal morphology of chondrocytes with a reduced ability to endure mechanical stress. The low amount of aggrecan protein in the ECM.	Xu et al., 2016 [173]
Neonatal-onset multisystem inflammatory disease (NOMID)	Patient 1: *NLRP3* c.1709A>G (p.Y570C) Patient 2: *NLRP3* c.919G>A (p.G307S)	The increased deposition of ECM and irregular endochondral ossification has been observed in mutant variants of chondrogenically differentiated iPSC. One of the potential causes was the upregulation of SOX9 in the cells mastered by the cAMP/PKA/CREB pathway.	Yokoyama et al., 2015 [174]
Metatropic dysplasia	*TRPV4* c.1812C>G (p.I604M)	Reduced expression of genes responsible for cartilage growth markers in chondrogenic micromasses studied derived from *TRPV4*-iPSC. The increased expression of COL1 and irregular mineralisation patterns during chondrogenesis indicate abnormal differentiation of chondrocytes.	Saitta et al., 2014 [175]
Fibrodysplasia ossificans progressiva (FOP)	*ACVR1* c.617G>A (p.R206H)	Enhanced expression of chondrogenesis related markers and production of ECM observed in FOP-iMSC.	Matsumoto et al., 2015 [176]
Hand Osteoarthritis	*GDF5* SNP: rs143383 SMAD3 SNP: rs12901499 *ALDH1A2* SNP: rs3204689 *IL1R1* rs2287047	Generated iPSC from patients with risk alleles correlated with the hOA exhibited decreased production of COL2 and proteoglycans in comparison with control	Castro-Viñuelas et al., 2020 [177]
Early-onset finger OA (efOA)	Genetic background not tested	The formation of vacuole-like structures was observed in chondrogenic masses formed in efOA with unknown aetiology. Compared to a healthy donor, the increased expression of hypertrophic markers and the secretion of cytokines and MMPS related with OA in chondrogenically differentiated efOA-iPSC.	Rim et al., 2021 [178]
Osteoarthritis	Artificial induced OA by addition IL-1β	The formation of mimicking osteochondral graft from iPSC cells enables us to observe changes during OA’s induction and study the biology of OA. This was shown by an increased expression of catabolic factors in constructed chips.	Lin et al., 2019 [179]

ACAN: aggrecan; ACGII: Achondrogenesis type II; ACH: Achondroplasia; ALDH1A2: Aldehyde dehydrogenase 1 family member A2; cAMP: 3′,5′-cyclic adenosine monophosphate; COL10A1: Alpha 1 type 10 collagen; COL2A1: Alpha 1 type II collagen; CREB: cAMP-response element-binding protein; CRISPR/Cas9: CRISPR-associated protein 9; ECM: extracellular matrix; efOA: early onset osteoarthritis; ER: endoplasmic reticulum; FGFR3:fibroblast growth factor receptor 3; FOCD: Familial Osteochondritis Dissecans; FOP: Fibrodysplasia ossificans progressiva; GDF5: Growth differentiation factor 5; HCG: Hypochondrogenesis; HCH: Hypochondroplasia; hOA: Hand Osteoarthritis; IHH: Indian hedgehog signaling molecule; IL-1β: Interleukin 1β; IL1R1: Interleukin 1 receptor, type I; iPSC: induced pluripotent stem cells; MATN3: Matrilin-3; MCDS: Metaphyseal chondrodysplasia type Schmid; MED: multiple epiphyseal dysplasia; MMP: metalloproteinases; NLRP3: NLR family pyrin domain containing 3; NOMID: Neonatal-onset multisystem inflammatory disease; PKA: cAMP-dependent protein kinase; SMAD3: SMAD family member 3; SNP: single-nucleotide polymorphism; SOX9: SRY-box transcription factor 9; SPD: Spondyloepiphyseal dysplasia; TD: Thanatophoric dysplasia; TRPV4: Transient receptor potential cation channel subfamily V member 4; UPR: unfolded protein response.

## Data Availability

Not applicable.
